# A pilot survey on the quality of life in respiratory rehabilitation carried out in COPD patients with severe respiratory failure: preliminary data of a novel Inpatient Respiratory Rehabilitation Questionnaire (IRRQ)

**DOI:** 10.1186/2049-6958-7-46

**Published:** 2012-11-20

**Authors:** Franco Pasqua, Annalisa Alesii, Katja Geraneo, Stefania Di Toro, Giuseppe La Torre, Antonella Sferrazza, Maria Grazia Mastrullo, Luigino Calzetta, Stefano Bonassi, Vittorio Cardaci, Alfredo Cesario

**Affiliations:** 1 Pneumology Rehabilitation, IRCCS San Raffaele Montecompatri, Rome, Italy; 2 Pneumology Rehabilitation, IRCCS San RaffaeleMontecompatri, Rome, Italy; 3 Institute of Hygiene, CatholicUniversity of the Sacred Heart, Rome, Italy; 4 Laboratory of Systems Approaches and Non Communicable Diseases, IRCSS San RaffalePisana, Rome, Italy; 5 Unit of Clinical and Molecular Epidemiology, IRCCS San Raffaele Pisana, Rome, Italy; 6 Scientific Direction, IRCSS San Raffale Pisana, Rome, Italy

**Keywords:** COPD, Inpatients, Quality of Life, Questionnaire, Respiratory rehabilitation

## Abstract

**Background:**

Measuring the state of health is a method for quantifying the impact of an illness on the day-to-day life, health and wellbeing of a patient, providing a quantitative measure of an individual’s quality of life (QoL). QoL expresses patient point of view by a subjective dimension and can express the results of medical intervention. Pulmonary rehabilitation is an essential component in the management of COPD patients, and measuring QoL has become a central focus in the study of this disease.

Although nowadays several questionnaires for measuring the QoL in COPD patients are available, there are no questionnaires specifically developed for evaluating QoL in COPD patients undergoing respiratory rehabilitation.

The aim of this study was to develop a novel questionnaire for the QoL quantification in COPD patients undergoing in-patient pulmonary rehabilitation program.

**Methods:**

The questionnaire, administered to COPD patients undergoing long-term oxygen therapy into a respiratory rehabilitation ward, was developed by a simple and graphic layout to be administered to elderly patients. It included one form for admission and another for discharge. It included only tips related to the subjective components of QoL that would be relevant for patient, although likely not strictly related to the respiratory function.

A descriptive analysis was performed for the socio-demographic characteristics and both the non-parametric Wilcoxon T-test and the Cronbach’s alpha index were calculated for evaluating the sensitivity of the questionnaire to the effects of respiratory rehabilitation and for identifying its consistency.

**Results:**

The physical and psychological condition of the 34 COPD patients improved after the rehabilitative treatment and this finding was detected by the questionnaire (overall improvement: 14.2±2.5%), as confirmed by the non-parametric Wilcoxon test (p<0.01). The consistency detected by the Cronbach’s alpha was good for both the questionnaire at admission and at discharge (0.789±0.084 and 0.784±0.145, respectively), although some items did not adequately measure the intended outcome.

**Conclusions:**

This proposed questionnaire represents a substantial innovation compared to previous methods for evaluating the QoL, since it has been specifically designed for hospitalized COPD patients undergoing respiratory rehabilitation with serious respiratory deficiency, allowing to effectively determining the QoL in these patients.

## Background

Measuring the state of health is a method for quantifying, in a standardized and objective manner, the impact of an illness on the day-to-day life, health and wellbeing of a patient. This process is very similar to gathering a well-structured clinical history but, instead of the collection of simple clinical findings, it provides a quantitative measure of the individual’s quality of life (QoL), which can be used for scientific purposes.

At present, chronic obstructive pulmonary disease (COPD) is recognized to be one of the major causes of death in industrialized countries and it represent a multisystemic pathology that induces disabilities and handicaps [1].

Pulmonary rehabilitation is an essential component in the management of COPD patients, and its success is mainly obtained through the improvement in exercise capacity, dyspnoea and QoL [2-8]. Measuring QoL has, thus, become a central focus in the study of COPD.

Traditionally, results in the fields of health/medicine and rehabilitation have almost always been measured through objective medical evaluations. On the other hand, recently there has been an ever-increasing focus on the patients’ perspective [9]. Therefore, the evaluation of outcomes patient-focused and measuring the wellbeing perceived by the individual in the physical, psychological, social and material areas [10,11], have lead to the development of a new concept of QoL [11,12] and of patient’s satisfaction. Indeed, the evaluation of the state of health and the influence of therapeutic intervention should incorporate not only changes in the gravity of illness, but also the impact upon the state of wellbeing.

The World Health Organization (WHO) defines QoL as “an individual’s perception of their position in life in the context of the culture and value systems in which they live and in relation to their goals, expectations, standards and concerns. It is a broad ranging concept, affected by the person’s physical health, psychological state, level of independence, social relationships, and to salient features of their environment” [11]. Nevertheless, different studies and reports on QoL used different definitions for QoL [10-12].

However, among all definitions, there is agreement concerning the concept that QoL expresses patient point of view by a subjective dimension and that QoL can express the results of medical intervention [2,10-12]. Therefore, the methods for evaluating QoL should be practical and simple to use, for clinical research and for evaluating the results of medical intervention.

Two main kinds of questionnaires for evaluating QoL exist, one generic and one illness-specific. The former includes the Sickness Impact Profile (SIP), the Nottingham Impact Profile (NIP) and the Short Form 36 (SF-36) [13-15]. The latter allows comparing patients suffering from different and specific pathologies and it investigates the characteristic aspects of an illness, including questions concerning symptoms linked to the illness itself. Effectively, a fair number of instruments/tools specific for COPD are at our disposal, including the Chronic Respiratory Questionnaire (CRQ), the St George Respiratory Questionnaire (SGRQ), the Maugeri Foundation Respiratory Failure Questionnaire, the Airways Questionnaire (AQ 30\20), the Breathing Problems Questionnaire (BPQ), the Pulmonary Functional Status & Dyspnea Questionnaire (PFSDQ) and the Pulmonary Functional Status & Dyspnea Questionnaire-Modified (PFSDQ-M) [16-22].

Nowadays, outcome measures are necessary for describing individual improvements and the efficacy of a rehabilitation program for COPD patients and consequently both CRQ and SGRQ represent the most used questionnaires for these patients, demonstrating to be responsive to respiratory rehabilitation [2,16,23,24]. Furthermore, due to the often incurable and relentlessly progressive respiratory deficiency led by COPD, the specific questionnaire named MRF-26 has recently been developed [18,21,22].

Therefore, the aim of the present study was to begin the development a novel questionnaire for the evaluation of QoL in patients suffering from respiratory failure due to COPD undergoing an in-patient pulmonary rehabilitation program.

## Methods

### Ethical approval and Consent

This study has been carried out in compliance with the Helsinki Declaration and it received the implied approval of the Ethical Committee of the Local Italian Health Authority “Azienda Sanitaria Locale” (ASL, reference number RM/H-05/2010). Furthermore, written informed consent was obtained from the patients for publication of this report and any accompanying images.

### Questionnaire characteristics

The questionnaire, built by a simple and graphic layout to be administered to elderly patients, included one form for admission and another for discharge. Since irrelevant matters for patients and topics that are not source of satisfaction probably would never influence the patient’s QoL, the questionnaire included only tips related to the subjective components of QoL that would be relevant for patient, although likely not strictly related to the respiratory function.

### Selection of items and score calculation

After a detailed comparison of all available questionnaires for respiratory diseases, items from the MRF-26 were chosen. Further items were chosen according to the clinical experience of patient responsiveness to the respiratory rehabilitation. Topics were hence modified in order to adhere to the core aims of our questionnaire and arranged for an easy compilation.

Both versions of the Admission Inpatient Respiratory Rehabilitation Questionnaire (Admission IRRQ) and Discharge IRRQ included 5 sections titled as follow sections: 1) “What symptoms have you got?”, a series of statements generally used by people with respiratory disorders to indicate the frequency of listed symptoms in the last month; 2) “How do you live?”, a series of statements related to normal activities, reporting how much the respiratory disorder(s) has limited those activities in the last month; 3) “My mood”, a series of statements which describe the person’s mood, reporting how often in the last month the patient felt the feelings described in the statements; 4) “How important is this to you?”, a list of sentences concerning different areas of life and how important was each specific area; 5) “What I think about the treatment I am having”, a list of statements concerning the pharmacotherapy and the treatment during the last month.

Section 1 included 8 items scored as: always = 1, often = 2, sometimes = 3, rarely = 4 and never = 5. Section 2 included 10 items scored as: always = 1, often = 2, sometimes = 3, rarely = 4 and never = 5 were assigned to items 1–6, 9 and 10. The items 7 and 8 were scored as: always = 5, often = 4, sometimes = 3, rarely = 2 and never = 1. Section 3 included 11 items scored as: always = 1, often = 2, sometimes = 3, rarely = 4 and never = 5. Section 4 included 14 items scored as: very important = 1, important = 2, somewhat important = 3, of little importance = 4, not important at all = 5. The last section 5 included one item scored as: true = 0, false = 1. The score output was low for poor QoL and high for a good QoL.

### Questionnaire administration and study population

The IRRQ was administered to COPD patients in ordinary admission regimen for respiratory failure undergoing long-term oxygen therapy (LTOT) to the Respiratory Rehabilitation ward of IRCCS San RaffaelePisana and San RaffaeleVelletri from January 2011 to December 2011.

The admission and discharge IRRQ was administered at time 0 (the day before the start of the rehabilitation cycle) and at time 1 (the day of discharge or on the day previous).

### Rehabilitation program

The program consisted of two daily sessions, 90 minutes each, five days a week for four weeks in Inpatient care and included: respiratory muscle training, strengthening of the abdominal walls and of both the upper and lower limbs, physical exercise training via the use of cycle ergometer, treadmill and arm ergometer, bronchial clearing techniques such as PEP-MASK, relaxation techniques and psychological and educational support.

### Statistical analysis

A descriptive analysis was performed for the socio-demographic characteristics. In particular, continuous variables were summarized with the median scores for nominal variables and both the absolute and percentage frequencies were reported. The two IRRQ questionnaires were summarized with the mode, expressed with the label and its frequency percentage, and with the count and corresponding frequency percentage of unanswered questions. For the 5^th^ section patients answered exclusively to questions pertinent to the type of treatment that they were undergoing (oxygen-therapy, ventilator, tracheostomy cannula).

In order to verify whether the questionnaire was sensitive to the effects of respiratory rehabilitation and, therefore, to assess the improvement or deterioration of the physical or psychological condition of patients after rehabilitative treatment, the non-parametric Wilcoxon T-test was conducted on the total scores obtained from the first 4 sections of the IRRQs.

The internal consistency of the questionnaire (sense/construction/meaning) was evaluated by calculating Cronbach’s alpha index for each of the 5 sections. In addition for each section, the likelihood of non-homogeneity of each item was evaluated with respect to all other items of that section. This analysis was performed by calculating the item-total correlation and the item-total correlation minus each-one item, in turn for all items of a single section.

It was not possible to carry out a combined analysis for the internal validity of the 5^th^ section since the patients responded exclusively to the questions which interested them personally. For this same reason, Cronbach’s alpha was calculated for two of three subsections because only two patients had both oxygen therapy and a tracheostomy tube. One patient doing all three therapies was included in the group of patients who underwent both oxygen therapy and mechanical ventilation. Bland and Altman [25] were taken as reference for the degree of accuracy of the Cronbach’s alpha index. They proposed that for scales which are used as research tools to compare groups, they may be less than in the clinical situation, when the value of the scale for an individual is of interest. To compare groups, values of 0.7 to 0.8 are regarded as satisfactory. On the other hand, for the clinical application, much higher values of alpha index are needed where the minimum is 0.90, and values > 0.95 are desirable.

Finally, the floor or ceiling effects have been considered to be present if more than 15% of participants achieved the lowest or highest possible score, respectively [26].

The statistical analysis was carried out separately for the questionnaire administered in admissions and for that administered at discharge, eliminating from the study -one by one- all those patients not responding to at least one question.

All analyses were carried out using the SPSS 12.00 for Windows statistics software package.

## Results

### Patients characteristics

COPD patients undergoing LTOT that voluntarily agreed to fill the IRRQ included 26 males and 8 females (total patients: 34) and the average hospital stay was 28±4 days. All patients were older than 65 years and with a Mini-Mental State Examination (MMSE) score higher than 18. About 68% of patients were married and retired and 62% needed assistance in completing the questionnaire. There were no patients with neuromuscular diseases and/or unstable hemodynamic conditions (recent myocardial infarction and unstable angina).

At the moment of admission, more than 50% of the subjects declared to have cough, morning dyspnoea (one person did not respond to the question), respiratory problems necessitating the intervention of the patient’s general practitioner (one person did not respond to the question), sleep disruption caused by cough or dyspnoea, sleepiness during the day.

The socio-demographic and clinical characteristics of patients that participated in the survey are summarized in Tables 1 and 2.

**Table 1:** Socio-demographic characteristics and method of compilation of the questionnaire

**Variables**	**N**	**%**
**Gender**		
Female	8	23.5
Male	26	76.5
**Marital Status**		
Divorced	1	2.9
Single	1	2.9
Widowed	9	26.5
Married	23	67.6
**Work**		
Employed	1	2.9
Unemployed	23	67.6
Retired	10	29.4
**Household companion**(**s**)		
Son/daughter	6	17.6
Spouse	22	64.7
None (Residing alone)	5	14.7
Other	1	2.9
**Method of compilation**		
Assisted compilation	21	61.8
Self compilation	13	38.2

**Table 2:** Clinical characteristics of the patients

**Parameters**	
Patients undergoing NIMV (n)	12
Patients with tracheostomy (n)	6
FEV_1_ (% predicted, mean±SD)	48.8±6.4
PaO_2_ (mmHg, mean±SD)	50.19±8.93
PCO_2_ (mmHg, mean±SD)	49.82±9.11

FEV_1_,Forced expiratory volume; NIMV, Non Invasive Mechanical Ventilation.

### General considerations on the questionnaire responses

Table 3 shows the most frequent category of response for each question of the questionnaire and the frequency of missing answers for each question. At admission, more than 50% of the patients had declared to have a serious shortness of breath while walking uphill or while climbing stairs, that their families always help them cope with their respiratory problems, that they do not feel oppressed by their families nor by those around them and that family support is very important to them. Finally, 67.6% of patients stated they were not bothered by receiving therapy in front of others.

**Table 3:** Responses to the questionnaire at both admission and discharge

**Item**			**Admission**		**Discharge**	
			**Mode (%)**	**Omissions n (%)**	**Mode (%)**	**Omissions n (%)**
**Section 1: What symptoms have you got**?						
1 I’ve had cough (with or without phlegm)			“Often” (26.5)	0	“Sometimes” (29.4)	0
2 I’ve been out of breath when I wake up in the morning			“Often” (32.4)	1 (2.9)	“Sometimes” / ”Never” (29.4)	0
3 I’ve been out of breath while resting			“Never” (35.3)	1 (2.9)	“Never” (47.1)	1 (2.9)
4 I’ve been out of breath while dressing, combing my hair, washing myself…			“Always” (38.2)	0	“Sometimes” (32.4)	0
5 I’ve been out of breath while walking uphill, while climbing stairs…			“Always” (52.9)	1 (2.9)	“Always” (35.3)	0
6 Because of my respiratory problems I have called my doctor			“Often” (32.4)	1 (2.9)	“Never” (42.4)	1 (2.9)
7 Cough or shortness of breath have disrupted my sleep			“Often” (32.4)	0	“Rarely” (35.3)	0
8 I have felt drowsy during the day			“Sometimes” / “Never” (32.4)	1 (2.9)	“Never” (29.4)	0
**Section 2: How do you live**?						
1 I’ve have difficulty doing anything at home (reading a book, receiving friends, listening to music…)			“Often” (41.2)	0	“Never” (38.2)	0
2 I have difficulty leaving the house to go shopping, go out with friends, pursue a hobby…			“Always” (38.2)	1 (2.9)	“Sometimes” (35.3)	0
3 Because of my respiratory disorder(s) I have felt like an invalid			“Often” / “Always” (32.4)	1 (2.9)	“Sometimes” (41.2)	0
4 My respiratorydisorder(s) have limited me in what I eat			“Never” (32.4)	0	“Never” (47.1)	0
5 My respiratory disorder(s) limit(s) my work activities			“Often” (38.2)	2 (5.9)	“Always” (29.4)	1 (2.9)
6 My respiratory disorder(s) limit(s) my sexual activity			“Often” (32,4)	4 (11.8)	“Always” (20.6)	5 (14.7)
7 My family helps me deal with my respiratory problems			“Always” (52.9)	1 (2.9)	“Always” (38.2)	1 (2.9)
8 The people around me help me deal with my respiratory problems			“Sometimes” (29.4)	0	“spesso” (38.2)	1 (2.9)
9 My family hinders me/oppresses me			“Never” (64.7)	1 (2.9)	“Never” (67.6)	0
10 The people around me hinder me/oppress me			“Never” (52.9)	2 (5.9)	“Never” (64.7)	0
**Section 3: My mood**						
1 I have felt sad			“Often” (41.2)	0	“Sometimes” / ”Never” (29.4)	1 (2.9)
2 I have cried without reason			“Sometimes” (32.4)	0	“Never” (67.6)	0
3 I have lost interest in doing the things I used to at home			“Often” (35.3)	2 (5.9)	“Never” (50.0)	2 (5.9)
4 I have lost interest in doing the things I used to outside home			“Often” (32.4)	1 (2.9)	“Never” (44.1)	2 (5.9)
5 I have lost interest in food			“Sometimes” (26.5)	0	“Never” (58.8)	1 (2.9)
6 I have trouble falling asleep			“Often” (26.5)	2 (5.9)	“Sometimes” (29.4)	1 (2.9)
7 I sleep fitfully			“Sometimes” (47.1)	0	“Sometimes” (35.3)	0
8 I feel tired and without energy			“Often” (35.3)	1 (2.9)	“Sometimes” (38.2)	0
9 I have been dissatisfied with myself, in what I do and how I behave			“Never” (26.5)	0	“Rarely” (32.4)	0
10 I have had difficulty concentrating, thinking, making decisions			“Never” (44.1)	1 (2.9)	“Never” (41.2)	1 (2.9)
11 I have wished I could die			“Never” (44.1)	0	“Never” (64.7)	0
**Section 4: How important is this to you**?						
1 At admission: Doing work around the house; at discharge: Reading			“Very important” (32.4)	0	“Important” (32.4)	1 (2.9)
2 Going out (going shopping, going out with friends, pursuing a hobby…)			“Very important” (38.2)	0	“Very important” (29.4)	1 (2.9)
3 My work activities			“Not important at all” (32.4)	2 (5.9)	“Very important” (29.4)	3 (8.8)
4 My sexual activity			“Not important at all” (29.4)	2 (5.9)	“Important” (35.3)	2 (5.9)
5 The support from my family			“Very important” (64.7)	1 (2.9)	“Very important” (64.7)	1 (2.9)
6 The support from people around me			“Very important” (50.0)	1 (2.9)	“Very important” (58.8)	0
7 The food I eat			“Very important” (38.2)	0	“Very important” (38.2)	0
8 My body image (being too thin or too fat)			“Very important” (35.3)	0	“Very important” (29.4)	1 (2.9)
9 Coughing			“Very important” (47.1)	2 (5.9)	“Very important” / ”Important” (32.4)	1 (2.9)
10 Shortness of breath in the morning			“Very important” (50.0)	3 (8.8)	“Very important” (29.4)	5 (14.7)
11 Shortness of breath while resting			“Very important” (38.2)	4 (11.8)	“Very important” / “Not important at all” (20.6)	3 (8.8)
12 Shortness of breath while walking uphill or climbing stairs…			“Very important” (64.7)	2 (5.9)	“Very important” (50.0)	1 (2.9)
13 Not being able to sleep because of shortness of breath			“Very important” (38.2)	3 (8.8)	“Very important” (26.5)	5 (14.7)
14 Being drowsy during the day			“Of little importance” (32.4)	4 (11.8)	“Not important at all” (29.4)	2 (5.9)
**Section 5: What do I think of the treatment I am having**?						
1 It bothers me to take medicine in front of others			“False” (67.6)	0	“False” (82.4)	1 (2.9)

The physical and psychological condition of patients after rehabilitative treatment improved from admission to discharge. In particular, the percentage of patients with shortness of breath when walking uphill, climbing stairs or with breathlessness in a “very important” manner, the percentage of patients that were not hindered by those around them, those patients that sometimes don’t sleep soundly and the percentage of patients that does not mind taking medicines in front of others improved overall of 14.2 ±2.5%. On the other hand, the situation became worse concerning to being helped in dealing with respiratory problems by family members (−14.7, 52.9% from admission to discharge).

Comparing the scores obtained for the four questionnaire sections of the two versions (admission and discharge) with the non-parametric Wilcoxon test, a significant statistical improvement (p<0.01) was observed for the first three sections, while significance (p=0.23) for section 4 was not attained (Table 4).

**Table 4:** Wilcoxon T-test for four sections of the questionnaire

**Section**	**Item number**	**Rank sum**	**p-value**
**What symptoms have you got**?			
Negative	3	22.00	<0.01
Positive	21	278.00	
Ties	5		
Total	29		
**How do you live**?			
Negative	4	14.00	<0.01
Positive	18	239.00	
Ties	4		
Total	26		
**My mood**			
Negative	5	43.00	0.01
Positive	19	257.00	
Ties	3		
Total	27		
**How important is this to you**?			
Negative	11	94.00	0.23
Positive	10	137.00	
Ties	2		
**Total**	23		

Age and gender did not had effect on the questionnaire response and none of the patients had the lowest or the higher possible score on the questionnaire, indicating that there was nor floor nor ceiling effect at both admission and discharge.

### Questionnaire at admission

The first section of the questionnaire presented 8 questions with the aim of evaluating patients’ symptoms. In this section data from 31 patients out of 34 were available because 3 (8.8%) patients did not answer to at least one question. Cronbach’s alpha demonstrated good internal consistency for the 8 questions (0.858). This index rose slightly (0.883) when the 5th and the last questions were eliminated (“I’ve been out of breath while walking uphill, while climbing stairs…” and “I have felt drowsy during the day”). Besides, this last question had a low correlation with the other items of the section (0.293).

In the second section of the questionnaire there were 10 questions which had the aim of evaluating if the respiratory deficiencies could limit the execution of some activities. In this section, data from 27 patients were considered because 7 (20.6%) did not answer to at least one question. Cronbach’s alpha showed a sufficient internal consistency for the 10 questions (0.754). The index, calculated on 27 patients, improved (0.845) when the last four questions were eliminated (“My family helps me deal with my respiratory problems”, “The people around me help me deal with my respiratory problems”, “My family hinders me/oppresses me”, “The people around me hinder me/oppress me”).

The third section of the questionnaire included 11 questions which had the objective of evaluating the mood of the person. In this section 30 patients answered to all questions, whereas 4 (11.8%) did not give at least one response. Cronbach’s alpha showed a good internal consistency of the 11 questions (0.857). This index rose slightly (0.867) when the last two questions were eliminated (“I have had difficulty concentrating, thinking, making decisions”, “I have wished I could die”).

The fourth section of the questionnaire presented 14 questions with the objective of evaluating how important each area of life was to each patient. In this section full data were available on 27 patients because 7 subjects (20.6%) did not answer to at least one question. Cronbach’s alpha did not show a good internal consistency for the 14 questions (0.687). The alpha value increased to indicate a good consistency (0.824) when a few questions were eliminated: “Doing work around the house”, “Going out”, “My sexual activity”, “The support from my family”, “The support from people around me”, “The food I eat”, and “My body image”.

In the 5^th^ section of the questionnaire there were 10 questions whose objective was to evaluate what patients think of the treatment they were having. Patients, however, responded exclusively to the questions which interested them, based on the type of treatment they were undergoing (oxygen therapy, ventilator with or without tracheostomy tube), with the exception of the first question to which everyone answered. One patient underwent a treatment with oxygen or a treatment with oxygen and ventilator, and either with or without a tracheostomy tube. As it was not possible, therefore, to carry out a combined analysis of internal consistency for this section, Cronbach’s alpha was calculated relatively to two of the three subsections, as only two patients were undergoing oxygen therapy via cannula.

There was then one further patient that carried out oxygen therapy with either ventilator or cannula, but he was included amongst the patients who practiced oxygen therapy and ventilator. For 21 patients who practiced only oxygen therapy (1 subject was excluded as he/she did not respond to all questions), Cronbach’s alpha (0.125) showed that the 4 items did not adequately measure the intended outcome. For 10 patients (one subject was eliminated because not responding to all the questions), who did either oxygen therapy or ventilator, the alpha value suggested an almost-good consistency (0.809). The index reached a good level (0.892) when the following items were eliminated: “My oxygen limits my day-to-day activities", “Oxygen is of little use to me”, “I find it embarrassing to be amongst people with oxygen” and “My ventilator is of little use to me”.

### Questionnaire at discharge

The questions of this questionnaire were exactly the same as those administered in admission, excluded for Section 4 in which the first question regarding the importance of “doing work around the house” was substituted with the importance of “reading”.

In the first section the number of patients considered in the study were 32 as 2 (5.9%) did not answer at least one question. Cronbach’s alpha demonstrated good internal consistency of the 8 questions (0.829), even though the value was slightly lower than that found in the same section at admission. This index rose slightly (0.852) when the 6^th^ question, “Because of my respiratory problems I have called my doctor”, was eliminated, considering the 33 patients who responded to all questions of the section as well as the remaining 7 items.

In the second section, 29 patients were analyzed, as 5 (14.7%) did not respond to at least one question. Cronbach’s alpha showed a low internal consistency for the 10 questions (0.569). This index rose slightly (0.647), and remained within the level of acceptability, when the 8^th^ question was eliminated: “The people around me help me deal with my respiratory problems”.

The third section, taking into account the 28 patients (82.4%) responding to all questions in the questionnaire, presented a Cronbach’s alpha of 0.857. The index rose very slightly (0.869) when two questions were eliminated: “I have been dissatisfied with myself, in what I do and how I behave” and “I have had difficulty concentrating, thinking, making decisions”.

In the fourth section, considering only 24 subjects (70.6%), Cronbach’s alpha was relatively high (0.881). The value became optimum when only for 4 of the 14 initial questions were considered: “Shortness of breath in the morning”, “Shortness of breath while resting, “Shortness of breath while walking uphill or climbing stairs…”, “Not being able to sleep because of shortness of breath”.

For the 5^th^ section of the questionnaire the Cronbach’s alpha was calculated exclusively for the subsection oxygen-therapy, as no patient had a tracheotomy tube and only 6 subjects used a ventilator and, therefore, data were available on 24 patients. The low Cronbach’s alpha (0.457) showed that the 4 items did not adequately measure the intended outcome. The index reached a value of 0.600 when the following items were eliminated: “My oxygen limits my day-to-day activities", “Oxygen is of little use to me”, “I find it embarrassing to be amongst people with oxygen”. Nevertheless, such a value does not suggest a good internal consistency.

## Discussion and Conclusions

Outcome measures are necessary for describing the individual improvement needed to confirm the efficacy of a therapeutic program, both pharmacological and rehabilitative. In COPD, that by definition is an incurable and progressive pathology, the measure of the state of the health, which correlates with the disease status, represents a fundamental moment. During the last years, different questionnaires have been developed with the aim of measuring the QoL in these patients, and some of these questionnaires are highly responsive to rehabilitation [2-8,21-23,25].

Nevertheless, the available questionnaires are limited as they are predominantly focused on COPD patients in a stable phase, scarcely taking into consideration inpatients, patients with serious respiratory deficiency and patients undergoing mechanical ventilation or tracheotomized. Effectively, it results complex to gather appreciable improvements in functional areas for these cluster of COPD patients,.

On the basis of these findings, our novel questionnaire includes substantial innovation as it has been designed for hospitalized patients and with serious respiratory deficiencies, undergoing oxygen therapy and/or mechanical ventilation and also via invasive measures.

The 44 items identified for the proposed questionnaire have been selected from the MRF-26, although further items have been chosen according to the clinical experience on the respiratory rehabilitation responsiveness of COPD patient with severe respiratory failure. Effectively, no questionnaires available in literature resulted sensitive enough to reveal modifications of the QoL in serious COPD patients, even mechanically ventilated and/or tracheostomized.

Our novel questionnaire is organized in two versions, one specific for admission and the other for discharge. These versions differ from each other since the questions administered at discharge refer explicitly to the period of hospitalization. In addition, compared to the most widely used questionnaires, our questionnaire dedicates much more attention to mood disorders, which are often present in COPD patients and frequently correlated with hypoxia. Furthermore, in accordance with an “approach based on necessity”, the new 4^th^ section included in this questionnaire proposed also questions on activities truly important to the patients and their QoL. Nevertheless, the novelty introduced with the 4^th^ section was associated with a low internal consistency, particularly at admission. However, the quality of the 4^th^ section significantly improved by eliminating few questions, including the “importance of doing work around the house” at admission and the “importance of reading” at discharge. Therefore, our novel questionnaire, and particularly the 4^th^ section, will undergo a deep revision during the next validation study by testing the proposed IRRQ on a validation population represented by the same cluster of COPD patients, but enhancing the population size.

A further characteristic of novelty for the proposed IRRQ is represented by the 5thsection, that exclusively refers to the specific therapy administered to each patient. Therefore, since the item of this section “What do I think of the treatment I am having” allowed comparing the feeling of different subjects that receive different treatment, we believe that this approach permitted to compare subjects undergoing a variety of treatment regimens.

Although the preliminary data of this novel questionnaire suggest that it fitted well on the study population, it remains essential to compare its effectiveness, and the putative superiority, with gold standard questionnaires most currently used. In any case, the comparison with others existing questionnaires will be complex mainly for the innovative characteristics of our questionnaire, that make it significantly different compared to the others. Effectively, the differences and innovations are noteworthy, as we administered the questionnaire both at admission and discharge to an extremely specific target population represented by hospitalized and serious COPD patients undergoing respiratory rehabilitation and, in addition, nowadays there is no gold standard questionnaire that selectively evaluates the QoL in the population cluster enrolled in our study. However, it might be of interest to challenge our novel questionnaire with gold standard in the same field in order to assay its efficacy in respiratory disease other than COPD, such as neuromuscular pathologies.

Finally, although the preliminary data of this study represent the initial development of a survey on the QoL in respiratory rehabilitation carried out in COPD patients with severe respiratory failure, our findings are promising and suggest that, after a validation study, the proposed IRRQ might determine the real QoL in patients suffering from respiratory failure due to COPD undergoing an in-patient pulmonary rehabilitation program.

## Competing interests

The authors declare that they have no competing interests.
